# Trimethylamine-N-oxide: a potential biomarker and therapeutic target in ischemic stroke

**DOI:** 10.3389/fneur.2023.1156879

**Published:** 2023-04-21

**Authors:** Yuan Liu, Juan Qu, Junjie Xu, Aiming Gu, Dezhi Deng, Xiaodan Jia, Baoxiang Wang

**Affiliations:** ^1^Jiaxing University Master Degree Cultivation Base, Zhejiang Chinese Medical University, Jiaxing, China; ^2^Department of Neurology, Affiliated Hospital of Jiaxing University, The First Hospital of Jiaxing, Jiaxing, China

**Keywords:** ischemic stroke, trimethylamine-N-oxide, gut microbiota, trimethylamine, stroke

## Abstract

Ischemic stroke is by far the most common cerebrovascular disease and a major burden to the global economy and public health. Trimethylamine-N-oxide (TMAO), a small molecule compound produced by the metabolism of intestinal microorganisms, is reportedly associated with the risk of stroke, as well as the severity and prognosis of stroke; however, this conclusion remains contentious. This article reviews the production of TMAO, TMAO’s relationship with different etiological types of ischemic stroke, and the possibility of reducing TMAO levels to improve the prognosis of ischemic stroke.

## 1. Introduction

Stroke is the second leading cause of death and the third foremost combined cause of death and disability worldwide; nearly 87% of strokes are ischemic (1). While treatment and secondary prevention of ischemic stroke has reduced ischemic stroke-related burden significantly (2), residual risks remain undiscovered. Therefore, identifying the potential contributing factors to ischemic stroke is critical to improving the management and treatment of ischemic stroke.

Growing evidence suggests that intestinal dysbiosis caused by altered diversity and an abundance in gut microbiota is associated with the pathogenesis, progression, and clinical outcomes of ischemic stroke (3). Trimethylamine-N-oxide (TMAO) is a low-molecular compound produced by gut microbiota via diet metabolization and is thought to be a “counteracting solute” that protects proteins from various destabilizing forces. Multiple studies have shown that changes in TMAO concentration predict the risk of stroke (4), as well as the severity (5) of stroke and its clinical outcome and mortality (6) in patients with ischemic stroke (7). This review examines the latest research progress on the generation of TMAO, TMAO’s relationship with different subtypes of ischemic stroke, and TMAO’s involvement in new treatment approaches for ischemic stroke.

## 2. TMAO generation

**Figure 1:**
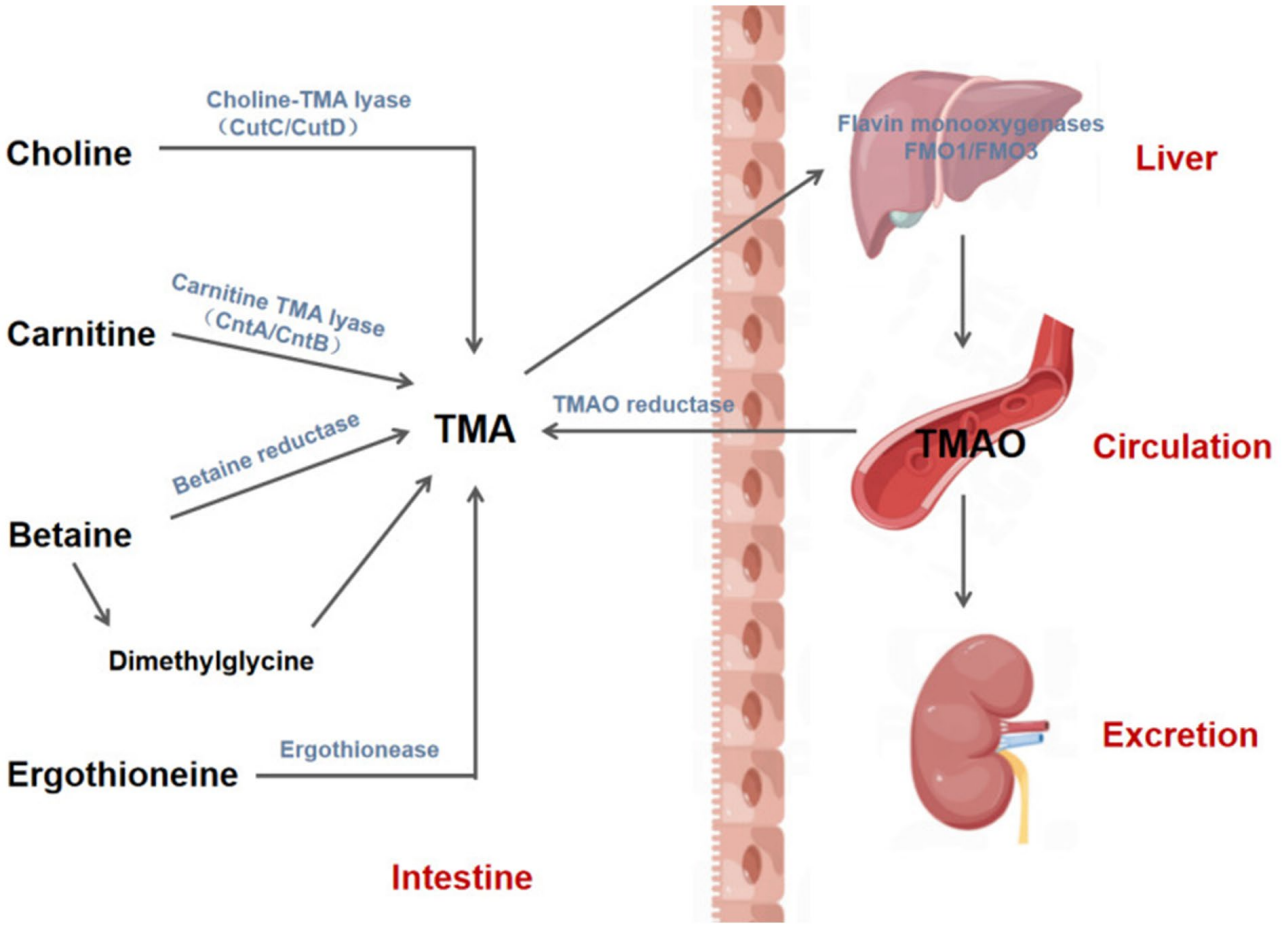
Biosynthesis and metabolism of TMAO. TMA is produced by gut mictrobiota through the action of various enzymes to metabolize phosphatidylcholine/choline, carnitine, betaine and ergothionein in the diet, and then absorbed by the intestine and transported to the liver through the portal vein, where TMA is further oxidized to TMAO by flavin monooxygenases 1 and 3 (FMO1/3). Afterwards, TMAO enters the circulatory system and is eventually excreted in the urine or reduced to TMA by TMAO reductase in the intestine. CutC/CutD, Choline trimethylamine-lyase system; CntA, A Rieske-type oxygenase protein; CntB, a predicted reductase with a plant-type ferridoxin domain; TMA, Trimethylamine; TMAO, Trimethylamine-N-oxide.

**Figure 2:**
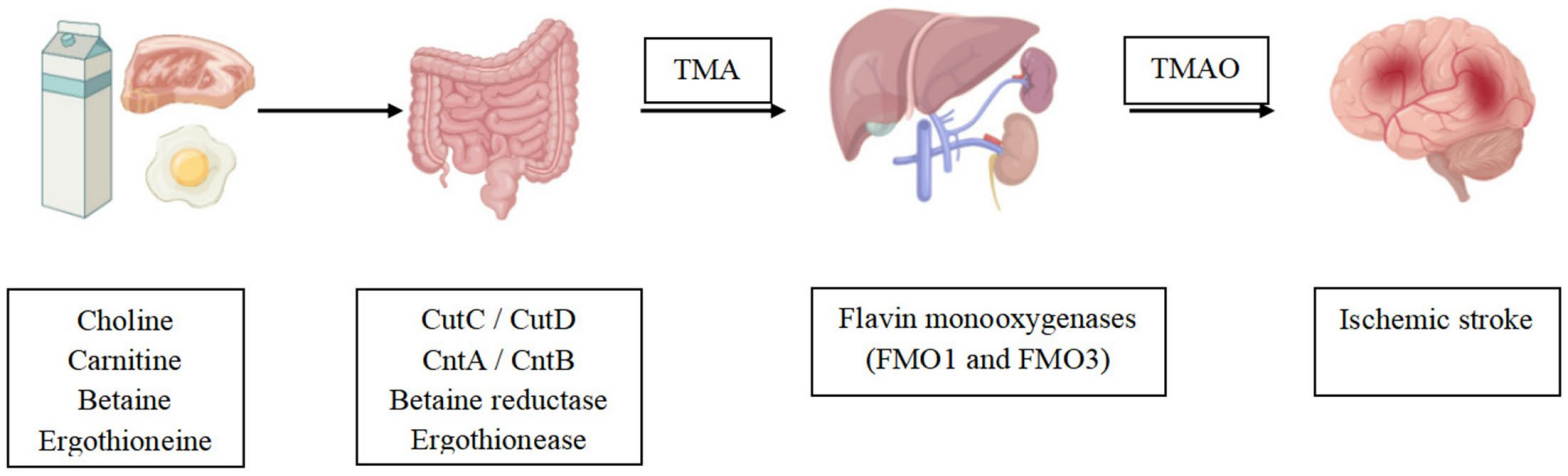
TMAO was shown to be associated with ischemic stroke events. Gut mictrobiota convert dietary nutrients into TMA, which is then oxidized to TMAO by the liver. Altered TMAO concentrations may play a potential role in ischemic stroke.

TMA, a precursor of TMAO, is formed by the intestinal flora through the metabolization of dietary compounds present in a diet. TMA’s core nutritional substrates include phosphatidylcholine/choline, carnitine, betaine, and ergothioneine. Choline, which is derived from foods, such as eggs, milk, and meat (red meat, poultry), is metabolized to Trimethylamine (TMA) by gut microorganisms using choline TMA lyase (e.g., CutC/CutD) (8). L-carnitine is found in high amounts in red meat, poultry, and some dairy products (9, 10) and is catalyzed by carnitine TMA lyase (e.g., CntA/CntB) to produce TMA (11). Betaine exists primarily in plants and is reduced to TMA directly by betaine reductase (8) or is converted indirectly to dimethylglycine, which is then metabolized to TMA via decarboxylation. Dietary sources of ergothioneine, like some soy foods and meat products (liver and kidney), are converted to TMA by ergothionease. Most of the TMA produced by intestinal microbial metabolism is absorbed by the intestinal tract and transported to the liver through the portal circulation, where it is further oxidized to TMAO by flavin monooxygenases (FMO1 and FMO3) and then released into the circulatory system. Eventually, approximately 1/2 of the formed TMAO is excreted, and the other 1/2 is reduced back to TMA via the action of TMAO reductase in the human gut (12) (Figures 1, 2).

## 3. TMAO and ischemic stroke

### 3.1. TMAO and the risk of ischemic stroke

There exists a positive correlation between circulating TMAO concentrations and the risk of ischemic stroke (13). Reportedly, the risk of stroke increases with a rise in TMAO levels: the risk of stroke in patients in the group with the highest concentration of TMAO was shown 34–68% higher than that of patients in the group with the lowest concentration (13). Similarly, there is a nonlinear association between elevated TMAO levels and the risk of stroke: the stroke risk correlation curve was shown to increase sharply at TMAO levels between 0 and 10 μmol/L; however, beyond this dose, the risk of stroke decreased slightly. Farhangi et al.’s (13) is also the first meta-analysis to establish a positive dose-dependent relationship between circulating TMAO concentration and the risk of stroke. The etiology of ischemic stroke is categorized according to the Trial of Org 10,172 in Acute Stroke Treatment (TOAST) classification (14). One assessment of the relationship between circulating TMAO concentration and the risk of ischemic stroke of different etiologies found that TMAO concentration correlated positively with the risk of aortic atherosclerotic (LAA) ischemic stroke, atrial fibrillation (AF)-caused cardiogenic stroke, and cerebral small vessel disease (CSVD). Chen et al.’s (15) analysis of the first case–control study of LAA stroke patients alongside asymptomatic patients (not at an acute disease state, based on self-report and the physical examination outcome) showed that Plasma TMAO concentrations in LAA stroke patients measured within 72 h of the onset of stroke were significantly higher than those in the normal control group, and this relationship persisted even after adjustments were made for age, sex, smoking, and other factors. Xu et al. (16) also reached a similar conclusion in a cross-sectional study of LAA stroke patients.

As a crucial risk factor for cardioembolic stroke, AF has been scrutinized. According to Liang et al.’s (17) results after evaluating 111 AF patients without ischemic stroke and 68 atrial fibrillation (IS-AF) patients with ischemic stroke, plasma TMAO levels in the IS-AF group were notably higher than those in the AF control group. This finding suggests that TMAO is an independent predictor of IS-AF and could improve ischemic stroke stratification in AF patients. An inquiry on CSVD conducted in 30 hospitals in China (18) found a link between TMAO and an increased risk of periventricular hyperintensity burden.

Although most studies have also established positive correlations between TMAO concentration and ischemic stroke, others have obtained different findings. A 4.8-year follow-up scrutiny of an elderly cohort at high cardiovascular risk in the Mediterranean (19) did not find a positive association between plasma TMAO concentrations and the risk of cardiovascular events (CVE), including stroke, myocardial infarction, and cardiovascular death. Yin et al. scrutinized blood samples from patients with LAA stroke and transient ischemic attack (TIA) within 24 h of their admission and reported drastically lower plasma TMAO levels in patients with LAA stroke and TIA than in the asymptomatic group (20). An analysis (21) of the plasma TMAO samples of 349 stroke patients from the Netherlands within 24 h of admission found that plasma TMAO levels were two times lower in stroke patients than in healthy controls. In Schneider et al.’s (22) research, plasma TMAO levels were markedly higher in patients with ischemic stroke than in the control group at the time of admission but decreased substantially 48 h after the stroke before increasing again in the subsequent 3-month follow-up. In contrast, TMAO levels in the control group did not change between admission and 48 h later. Schneider et al. further emphasized the importance of the time course of circulating TMAO concentrations in ischemic stroke and suggested that TMAO levels are best measured during the acute phase (<24 h), which is consistent with results from Tan et al. (23) on a Chinese acute ischemic stroke population.

Based on the current conflicting data on the specific role of TMAO in ischemic stroke patients, the concentration of TMAO in patients with ischemic stroke is possibly time-dependent. However, an unstandardized TMAO measurement time may provide a partial explanation for the differences in TMAO concentration in patients with ischemic stroke; some studies have not even reported TMAO measurement times. At the same time, a reduction in TMAO levels in patients with an ischemic stroke within 7 days of the onset of the condition could also be caused by treatment, especially treatment with antiplatelet drugs (24). In explaining their results in the follow-up examination of the elderly in the Mediterranean, the authors pointed to the low intake of meat and meat products in the Mediterranean diet and the synergistic effect of nutrient-rich foods, such as vegetables, fruits, legumes, etc., as jointly promoting favorable changes in the intermediate pathways of cardiometabolic risk, including blood lipids, Insulin sensitivity, oxidation, inflammation, and vasoreactivity (23), ultimately leading to a considerably reduced risk of ischemic stroke. Because existing publications have only demonstrated correlations between TMAO levels and the risk of ischemic stroke, the exact causal relationship and the impact of different treatment options for patients with ischemic stroke and previous stroke on TMAO levels must be deciphered further.

### 3.2. TMAO and the subtypes of ischemic stroke

The relationship between TMAO levels and ischemic strokes of different etiologies (according to the TOAST classification) is currently unclear. In Schneider et al.’s (22) prospective comparison of plasma TMAO levels in 196 patients with ischemic stroke according to the etiology of stroke (according to the TOAST classification), the correlation between TMAO levels and the number of vascular risk factors was weak, and there was no significant association between stroke subtypes. In addition, the difference in median TMAO levels between patients with LAA type and cardioembolic types was not statistically relevant. Another study obtained comparable results (25). The above phenomena are possibly due to overlapping risk factors among patients with ischemic stroke. Patients with cardioembolic stroke also suffer from hypertension (26), diabetes (27, 28), renal insufficiency (29–31), and coronary artery disease (32), disorders that can all lead to elevated plasma TMAO concentrations, resulting in smaller differences in plasma TMAO levels among stroke patients with different etiologies. Xu et al. (33) found drastically higher plasma TMAO levels in stroke patients in the LAA group and CE (Cardioembolic) group than in the control group, speculatively pointing to TMAO as crucial to the pathophysiology of atherosclerosis-related stroke and suggesting that the increase in TMAO levels in the CE group was probably associated with the ability of TMAO to promote platelet hyperresponsiveness and heighten the risk of thrombosis (34). This study partly explained the lack of any significant differences in TMAO concentrations among different subtypes. Additional research on the causal relationship leading to this phenomenon is required.

### 3.3. TMAO and the severity of ischemic stroke

Using a rat model of middle cerebral artery occlusion/reperfusion (MCAO/R), Su et al. (35) found TMAO to promote reactive astrogliosis and glial scar formation by inhibiting Smurf2 and upregulating ALK5 to prevent Neurological recovery; neurological recovery was markedly worse in MCAO/R rats treated with TMAO compared to MCAO/R rats. A clinical study on the first acute ischemic stroke (36) diagnosed 97 (26.8%) out of 362 patients with early neurological deterioration (END) and reported higher plasma TMAO levels in END patients, establishing a dose–response relationship between the two. Another case–control study on the first acute ischemic stroke in China (37) reached a similar conclusion and established a positive correlation between serum TMAO level and the NIHSS score. In Li et al.’s (27) cross-sectional study of 108 type 2 diabetes mellitus (DM) patients with ischemic stroke, patients with moderate to severe stroke (NIHSS score > 5) had higher plasma TMAO levels than those with mild stroke (NIHSS score ≤ 5); that is, higher plasma TMAO levels were associated with stroke severity. In these animal experiments (38) and clinical studies (5, 6, 15), elevated circulating TMAO levels upon admission are independent predictors of END and stroke severity in patients with acute ischemic stroke.

### 3.4. TMAO and the prognosis of ischemic stroke

Little is known about the relationship between TMAO concentrations and ischemic stroke outcomes, including post-stroke infections, cognitive impairment, adverse functional outcomes, and mortality. In an observational case–control study (21), Haak et al. found that stroke and post-stroke infection were associated with severe disturbances in the gut microbiota profile, and this was manifested to a greater degree by the enhanced presence of bacteria linked to TMAO production and the reduction in butyrate-producing bacteria. An animal study on post-stroke cognitive impairment (PSCI) showed that (39) TMAO promotes neuronal aging, damages synapses, and downregulates the expression of synaptic plasticity-related proteins and mTOR signaling pathways, thereby accelerating brain aging and cognitive decline in mice. TMAO has also been revealed to upregulate macrophage scavenging receptors and induce the expression of CD68, a marker with connections to cognitive impairment (40). According to reports from clinical studies, elevated plasma TMAO levels in patients with acute ischemic stroke are linked to PSCI, as determined using MoCA score (41) and MMSE score (42). Tu et al. (7) advanced TMAO as a probable new target for early prediction and treatment of stroke and vascular cognitive impairment. Zhong et al. (43) identified a weak relationship between TMAO levels and MMSE-defined cognitive impairment and no significant association between TMAO and MoCA-established cognitive impairment in patients with acute ischemic stroke at the 3-month post-stroke follow-up mark. Reportedly, higher plasma TMAO concentrations in patients with ischemic stroke predict higher adverse functional outcome events and mortality (6, 44); baseline TMAO levels are considerably more abundant in patients with 90 days or 12 months critical ischemic events and unfavorable functional outcomes (23); and baseline TMAO levels improve the prognostic accuracy of NIHSS scores and conventional risk factors for major ischemic events at 90 days.

Based on the aforementioned studies, aberrations in gut bacteria producing trimethylamine and butyrate are independent predictors of an increased risk of infection after stroke. However, the relationship between TMAO concentration and PSCI remains debatable and could have something to do with the fact that some reported trials have not excluded patients with cognitive decline before stroke. Also, differences in the length of follow-up for stroke patients may lead to false-negative in some patients, thus affecting conclusions. Nevertheless, the finding that elevated early TMAO levels predict poor stroke outcomes broadens the possibility of using TMAO clinically as an independent prognostic marker and therapeutic target.

### 3.5. TMAO and the recurrence of ischemic stroke

Wu et al.’s (45) analysis of the plasma TMAO levels of 268 patients undergoing carotid artery stenting (CAS) found significant associations between high levels of TMAO and the risk of stroke recurrence. Haghikia et al. (46) reached a similar conclusion in their prospective cohort study: that is, the group with the highest concentration of plasma TMAO was more prone to recurrent stroke within 1 year of follow-up than the group with the lowest concentration. The risk of CVE was dose-dependent. Another investigation demonstrated that the risk of recurrence of major vascular events increased by 2.128 times in ischemic stroke patients with plasma TMAO levels higher than 126.83 pg/mL, (15) a finding consistent with results from a recent observational trial (47), providing a new rationale for the prevention of secondary ischemic strokes.

## 4. Treatment and intervention

### 4.1. Diet

Diet is a key factor affecting the concentration of TMAO, with long-term dietary habits having a non-negligible impact on the ability to synthesize TMAO in humans and mice. As has been revealed previously, a high-salt, high-fat, and red-meat-rich diet can amplify circulating TMAO levels (12, 48, 49), omnivores produce more TMAO – through a microbiome-dependent mechanism – than vegans/vegetarians after the ingestion of L-carnitine. Additionally, consuming Mediterranean or vegetarian diets results in the effective reduction of the production of TMAO. As a food ingredient, 3,3-Dimethyl-1-butanol (DMB) (50) diminishes TMAO levels by inhibiting the action of TMAO lyase, which lessens TMA production. Resveratrol (RSV) (51) hinders the production of TMA by reshaping the microbiota; that is, increasing the relative abundance of Bacteroides, Lactobacilli, and Bifidobacteria, thereby reducing TMAO levels. Therefore, a change in dietary habits can result in an decrease in TMAO levels.

### 4.2. Movement

Allegedly, (52) sedentary and light physical activity have not been found to be associated with TMAO. Meanwhile, moderate to vigorous physical activity (MVPA) correlates negatively with TMAO levels. MVPA can reduce TMAO levels by 0.584 μmol/L for 30 min every day. This result suggests that exercise intensity may also be important. A possible mechanism for this is MVPA’s potential help in promoting a healthier gut microbiota environment that is less conducive to TMAO generation (53, 54).

### 4.3. Probiotics

According to findings from studies with animals, probiotic supplementation can reduce TMAO concentrations; however, the same results have not been obtained in clinical trials (55–58). Enterococcus faecium WEFA23 (59) is a potential probiotic strain in Chinese infants with the ability to decrease TMAO production by downregulating the transcription levels of flavin monooxygenase 3 and remodeling the gut microbiota. *Lactobacillus plantarum* ZDY04 (60) and *Lactobacillus casei* (61) can reshape the composition of intestinal flora by regulating the abundance of Lactobacillus and Bacteroidetes to lessen the concentration of TMAO. However, several other clinical trials have established that probiotic supplementation does not reduce TMAO concentrations. In conclusion, the impact of probiotics on TMAO is inconclusive and more clinical trials are needed to decipher this conundrum.

### 4.4. Antibiotics

The use of broad-spectrum antibiotics has been shown to reduce the risk of infection in stroke patients, as well as suppress plasma TMAO levels (62), with this inhibitory effect disappearing after the discontinuation of the antibiotics. The adverse consequences of the long-term use of antibiotics, including susceptibility to infection, drug allergy, antibiotic resistance, and the occurrence of antibiotic-related metabolic diseases, also constitute issues that cannot be ignored. Therefore, using antibiotics clinically to suppress plasma TMAO levels is a practice that must yet be scrutinized even further.

### 4.5. Integrative Chinese and Western medicine

**Figure 3:**
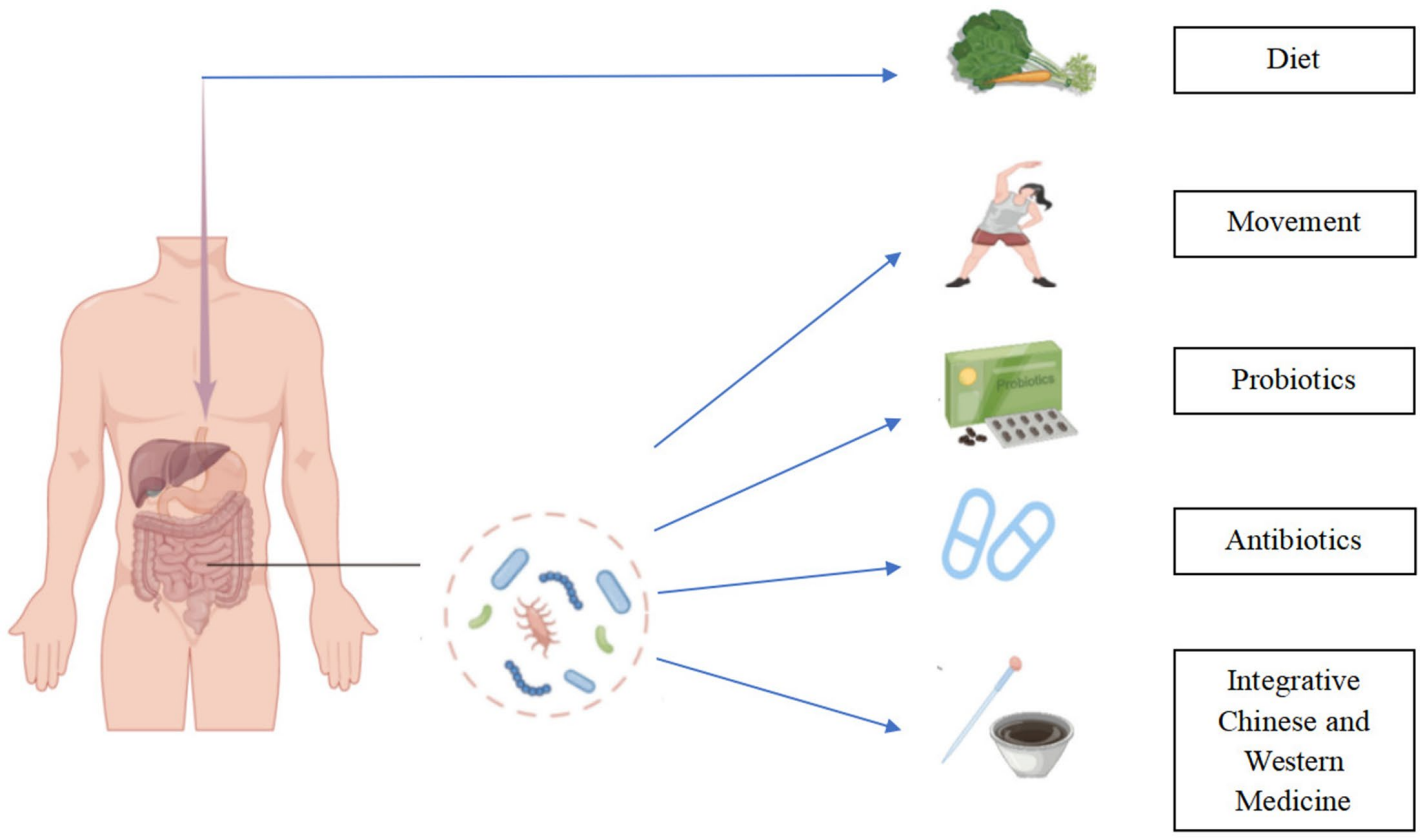
Regulating TMAO in the treatment of ischemic stroke. Modification of diet, rigorous exercise, rational use of probiotic supplements and antibiotics, and integration of traditional Chinese and Western medicine can reduce the burden of ischemic stroke by reducing TMAO levels.

Another mechanism of TMAO presence involved in acute ischemic stroke is inflammatory response. Inflammatory activities after the onset of acute ischemic stroke can further promote brain injury and lead to the poor prognosis of acute ischemic stroke. The Sanhuang Xiexin decoction and Tanhuo decoction have significant anti-inflammatory effects (63). Integrated traditional Chinese and Western medicine treatment [SX (Sanhuang Xiexin decoction + Western medicine), ITCM (Tanhuo decoction + Western medicine), Naochang Tongtiao acupuncture + Western medicine, berberine] have been shown to reduce TMAO levels. SX, ITCM, and Naochang Tongtiao acupuncture + Western Medicine treatment can equally improve the neurological function of patients with acute ischemic stroke (64–68), as demonstrated by the drastically diminished incidence of cerebrovascular events in patients treated with SX and ITCM over 3 and 6 months. In a nutshell, the combination of traditional Chinese medicine with Western medicine can enhance the impact of therapy on ischemic stroke patients to a certain extent, and this sort of impact is that which is a new basis for the treatment of stroke (Figure 3).

## 5. Summary

This article reviews the relationship between TMAO and stroke of different etiologies, as well as the possible treatment options for reducing circulating the concentration of TMAO. Unfortunately, most available studies are correlational studies, and figuring out whether changes in circulating TMAO levels are a cause or a consequence of ischemic stroke remains a challenge. Moreover, reducing the concentration of TMAO to an appropriate target value to treat stroke is surrounded by controversy (13, 69). Further research is many aspects is, therefore, of significant necessity.

Still, as revealed in this review, TMAO, as a derivative of the gut microbiota, is an important player in ischemic stroke. Investigations have uncovered the marker property of TMAO in predicting the incidence, progression and prognosis of ischemic stroke. Novel treatment approaches to mitigate the burden of ischemic stroke by reducing TMAO levels include adjusting diet, exercising rigorously, using probiotic supplements, and integrating traditional Chinese with western medicine. Antibiotics can inhibit the production of TMAO effectively, but the side effects of their long-term use should be factored into deciding whether to use them or not.

While researchers have proposed a new treatment concept to improve the prognosis of ischemic stroke patients by cutting TMAO levels, there is currently a lack of evidence to support that concept; therefore, the feasibility of bringing this concept to a widely applicable fore rests on additional in-depth experimentations.

## Author contributions

All authors listed have made a substantial, direct, and intellectual contribution to the work and approved it for publication.

## Conflict of interest

The authors declare that the research was conducted in the absence of any commercial or financial relationships that could be construed as a potential conflict of interest.

## Publisher’s note

All claims expressed in this article are solely those of the authors and do not necessarily represent those of their affiliated organizations, or those of the publisher, the editors and the reviewers. Any product that may be evaluated in this article, or claim that may be made by its manufacturer, is not guaranteed or endorsed by the publisher.
